# The Effect of Metformin on Survival Outcomes of Non-Metastatic Breast Cancer Patients with Type 2 Diabetes

**DOI:** 10.31557/APJCP.2021.22.2.611

**Published:** 2021-02

**Authors:** Bita Behrouzi, Mohammad Zokaasadi, Mohhamad Ali Mohagheghi, Amir Hosein Emami, Sanambar Sadighi

**Affiliations:** 1 *Geisel School of Medicine at Dartmouth, Hanover, NH, USA. *; 2 *Hematology, Oncology and Stem Cell Transplantation Research Center, Tehran University of Medical Sciences, Tehran, Iran. *; 3 *The Cancer Research Center of the Cancer Institute, Tehran University of Medical Sciences, Tehran, Iran. *; 4 *Department of Hematology-Oncology, Cancer Institute of Iran, Tehran University of Medical Sciences, Tehran, Iran. *

**Keywords:** Breast neoplasms, survival, metformin

## Abstract

**Background:**

There are still inconsistencies about the role of metformin on breast cancer. This study was designed to assess metformin’s effect on the prognosis of female breast cancer patients with type II diabetes.

**Methods:**

The present research was carried out as a retrospective cohort study between 2003 and 2014. Breast cancer patients with pre-existing type II diabetes mellitus were included. Overall survival (OS) and relapse-free survival (RFS) were measured as the main endpoints. Kaplan-Meier estimate was used to calculate survival rates**.**

**Results:**

217 patients were included with a mean age of 53.32±11.10 years. 148 (68.2%) patients were prescribed metformin and 69 (31.8%) took other antidiabetic drugs (non-metformin group). Five-year OS and RFS rates for all patients were 82.5% (95% CI: 76.0%-87.4%) and 71.1% (95% CI: 64.2%-77.0%) respectively. Log-rank test showed that the metformin group had a significant advantage over the non-metformin group in terms of both OS and RFS rates (P<0.001 for both). Five-year OS and RFS rates for metformin group were 91.9% (95% CI: 85.4%-95.6%) and 82.8% (95%CI: 75.5%-88.2%) respectively; the same rates for non-metformin group were 59.1% (95% CI: 43.9%-71.5%) and 39.3% (95%CI: 25.1%-53.1%) (P<0.001 for both). Results of proportional hazards model, after adjustment for body mass index, age, and tumor stage, depicted an independent prognostic value of metformin use with multivariate hazard ratio of 0.15 (95% CI: 0.07-0.32) for OS and 0.23 (0.14-0.40) for RFS compared to non-metformin group (P<0.001 for both).

**Conclusion:**

This study indicated that using metformin for diabetic breast cancer patients is associated with favorable results regarding recurrence and survival rates.

## Introduction

Breast cancer is the most common cancer in females around the globe causing a substantial number of deaths annually (Siegel et al., 2019). Based on the Global Cancer Observatory report, in 2018, breast cancer occupied the first rank as the most frequent type of cancer and the second most common cause of cancer death among the whole population (Bray et al., 2018). It is also the most frequent cancer of Iranian women with an age-standardized ratio of 33.2 breast cancer cases per 100,000 (Nafissi et al., 2018). Diabetes mellitus (DM) is a major health problem in Iran and globally, with its prevalence in the Iranian adult population reaching 11.4% based on recent investigations (Esteghamati et al., 2017). Notably, statistics on DM show a growing trend compared to the previous decade (Esteghamati et al., 2017). There is evidence supporting that DM patients are at increased risk of developing more invasive forms of cancer (Gong et al., 2016) and that diabetic breast cancer patients have a worse outcome compared to non-diabetics (Peairs et al., 2011; Behrouzi et al., 2017). Metformin, a biguanide antidiabetic agent used as a first-line therapy for type 2 DM particularly in overweight and obese patients, has recently come back into the realm of translational and clinical oncology research due to its potential effects on reduction of cancer incidence (Chlebowski et al., 2012) as well as its synergistic effects on cancer treatment (Chuang et al., 2018; Yu et al., 2019). This impact has been examined in a vast majority of solid tumors (e.g. colorectal, pancreas and lung) (Dowling et al., 2011; Ramjeesingh et al., 2016; Xu et al., 2018; Zhang et al., 2018; He et al., 2019). Both in vitro and in vivo studies on the role of metformin on breast cancer have been performed but have not reached a consistent result. The metformin effect on breast cancer still remains controversial (Zakikhani et al., 2006; Vazquez-Martin et al., 2011; Lega et al., 2013; Oppong et al., 2014). Therefore, we conducted a retrospective study to evaluate the effect of metformin in a cohort of diabetic breast cancer patients.

## Materials and Methods

From July 2003 to February 2014, diabetic patients undergoing treatment for breast cancer at the Cancer Institute of Iran were chosen for this study. Inclusion criteria were stages I to III breast cancer at the time of diagnosis and pre-existing type II DM. Exclusion criteria were breast cancer with distant metastasis, gestational DM, steroid-induced DM, type I DM and male breast cancer. All patients received chemotherapy and/or hormonal therapy. The patients were followed until death or December 2016. Since some limitation in research budgets we were not able to prolong the follow up period beyond the end of 2016. DM type II diagnosis was confirmed by blood glucose testing and self-report. The antidiabetic medication was extracted from the medical files and the patients were categorized into one of the two groups: metformin users, comprising those who had took at least 1000 milligrams of metformin during the follow up period and non-metformin group, including those used other antidiabetic drugs. The study protocol was approved by Ethics Committee of our institution.

TNM staging based on the American Joint Committee on Cancer’s 2020 manual was used for staging the tumors. Hormonal status of the samples for estrogen and progesterone receptors (ER and PR) were tested by immunohistochemistry (IHC). A sample was confirmed as HER2 positive once one of the following criteria were met: three positive overexpression in IHC was seen or Fluorescent in Situ Hybridization (FISH) amplification was observed. Tumor subtypes were categorized based on hormonal and HER2 status: luminal A (ER positive and/or PR positive, HER2 negative), luminal B (ER positive and/or PR positive, HER2 positive), HER2 enhanced type (ER negative, PR negative, HER2 positive), and triple-negative (ER negative, PR negative, HER2 negative). All specimens were rechecked for the purpose of the research. 

Differences were tested using the Chi-square test for categorical variables and the Independent Samples t-Test for continuous variables. Overall survival (OS) was defined as the time from pathologically confirmed diagnosis to all-cause death or end of the follow-up time. Recurrence-free survival (RFS) time was defined as the time from diagnosis to the first local, regional or distant recurrence of the disease. Kaplan-Meier estimate was used to calculate the survival rates. The difference between metformin and non-metformin groups was compared by the log-rank method. Cox regression analysis (uni- and multivariate analysis) was used to assess the effect of variables on survival outcomes. Tests were 2-sided and P-values of less than 0.05 were considered significant. Analyses were performed by R software for windows version 3.5.1 (survival package) and IBM SPSS statistics version 23. 

## Results

A total of 217 female diabetic patients were included in the study. Mean age at diagnosis was 53.32±11.10 years. Amongst patients, 148 were treated by metformin (68.2%) and 69 (31.8%) used other antidiabetic drugs. There was no significant difference between metformin and non-metformin groups regarding age, weight, height, body mass index (BMI), hormone receptor status, tumor subtype, chemotherapy protocol and tumor stage. Basic characteristics of the studied patients were summarized in Table 1. 

During a median follow-up time of 60 months (ranging from 1 to 160 months), a total of 37 deaths occurred, of which 11 (29.73%) were in the metformin group and the remaining 26 (70.27%) were in the non-metformin group (P<0.001). Mortality rates were 7.43% for the metformin group and 37.68% for the non-metformin group. Kaplan-Meier estimate for all patients showed 1- and 5-year OS rates of 97.7% (95% CI: 94.5%-99.0%) and 82.5% (95% CI: 76.0%-87.4%) respectively. The number of recurrences was significantly lower in the metformin group with 28 recurrences in the metformin group compared to 35 recurrences in the non-metformin group (P<0.001). Relapse rates were 18.92% for the metformin group and 50.72% for the non-metformin group. RFS rates for the 1st and 5th years were 94.4% (95% CI: 90.3%-96.8%) and 71.1% (95% CI: 64.2%-77.0%) respectively for all patients. Log-rank test revealed that the metformin group had significantly superior OS and RFS rates. Results of the log-rank tests are summarized in Table 2. 

**Figure 1:**
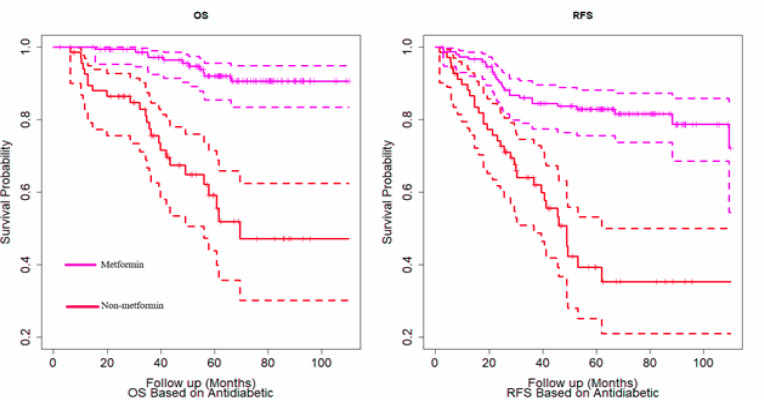
OS (Overall Survival) and RFS (Recurrence-Free Survival) Curves (Solid Line) for Metformin and Non-Metformin Groups with Corresponding 95% Confidence Intervals (Dashed Lines)

5-year OS rates in the metformin and the non-metformin group were 91.9% (85.4%-95.6%) and 59.1% (43.9%-71.5%) while the RFS rates were 82.8% (75.5%-88.2%) and 39.3% (25.1%-53.1%) respectively (P<0.001 for both). These significant differences were shown in Figure 1.

The proportional hazards model presented three prognostic factors affecting OS and RFS: tumor stage, BMI and use of metformin. After adjusting for age, tumor stage and BMI, metformin usage resulted in 85% lower risk of death and 77% lower risk of relapse. Hazard ratio was 0.15 (95% CI: 0.07-0.32; P<0.001) for OS and 0.23 (0.14-0.40; P<0.001) for RFS respectively compared to the non-metformin group. Results of the Cox proportional hazards model were summarized in Table 3.

**Table 1:** Basic Characteristics of Studied Population

Variable	Total (N=217)	Non-metformin (N=69)	Metformin (N=148)	P
Age at diagnosis, years				
Mean ± SD	53.32±11.10	55.22±11.20	52.44±10.98	0.09
Body weight, Kg				
Mean ± SD	74.10±13.78	75.21±14.04	73.59±13.67	0.43
Height, cm				
Mean ± SD	154.45±6.63	153.66±6.31	154.81±6.76	0.24
Body Mass Index, kg/m^2^				
Mean ± SD	31.13±5.80	31.96±6.29	30.75±5.54	0.16
< 35 n(%)	167 (79.15%)	48 (71.64%)	119 (82.64%)	0.07
≥ 35 n(%)	44 (20.85%)	19 (28.36%)	25 (17.36%)	
ER				
Negative n (%)	65 (30.1%)	24 (35.29%)	41 (27.70%)	0.27
Positive n(%)	151 (69.9%)	44 (64.71%)	107 (72.30%)	
PR				
Negative n (%)	79 (36.57%)	29 (42.65%)	50 (33.78%)	0.23
Positive n (%)	137 (63.43%)	39 (57.35%)	98 (66.22%)	
HER2				
Negative n (%)	170 (79.07%)	56 (82.35%)	115 (77.70%)	0.48
Positive n (%)	45 (20.93%)	12 (17.65%)	33 (22.30%)	
Subtype				
Luminal-A n (%)	131 (60.93%)	40 (58.82%)	91 (61.90%)	0.45
Luminal-B n (%)	19 (8.84%)	4 (5.88%)	15 (10.20%)	
Triple-Negative n (%)	39 (18.14%)	16 (23.53%)	23 (15.65%)	
HER2type n (%)	26 (12.09%)	8 (11.76%)	18 (12.24%)	
Chemotherapy				
Adjuvant n (%)	196 (90.32%)	59 (85.51%)	137 (92.57%)	0.14
Neo-adjuvant n (%)	21 (9.68%)	10 (14.49%)	11 (7.43%)	
TNM stage				
I n(%)	35 (16.59%)	9 (13.64%)	26 (17.93%)	0.66
IIA n(%)	55 (26.07%)	14 (21.21%)	41 (28.28%)	
IIB n(%)	46 (21.80%)	16 (24.24%)	30 (20.69%)	
IIIA n(%)	44 (20.85%)	14 (21.21%)	30 (20.69%)	
IIIB n(%)	14 (6.64%)	6 (9.09%)	8 (5.52%)	
IIIC n(%)	17 (8.06%)	7 (10.61%)	10 (6.90%)	

ER, estrogen receptor; PR, progesterone receptor

**Table 2:** Differences of OS and RFS Based on Prognostic Factors

Covariate		5-year OS (95% CI)	P	5-year RFS (95% CI)	P
Antidiabetic	Metformin	91.9 (85.4-95.6)	<0.001	82.8 (75.5-88.2)	<0.001
	Non-metformin	59.1 (43.9-71.5)		39.3 (25.1-53.1)	
Subtype	Luminal A	83.6 (75.1-89.4)	A0.8	71.9 (62.8-79.1)	0.9
	Luminal B	77.9 (35.4-94.2)		71.5 (43.8-87.2)	
	HER2 type	79.3 (57.1-90.9)		77.1 (52.4-90.1)	
	TNBC	80.6 (63.6-90.3)		67.2 (49.4-79.9)	
Age, years	>50	79.3 (70.0-86.0)	0.3	77.6 (66.3-85.4)	0.3
	≤50	86.6 (76.5-92.6)		66.6 (57.2-74.4)	
BMI, kg/m^2^	≥35	62.9 (45.7-76.0)	<0.001	53.7 (37.2-67.7)	0.005
	<35	88.2 (81.4-92.7)		76.2 (68.4-82.2)	
TNM Stage	I	96.4 (77.2-99.5)	<0.001	97.1 (80.9-99.6)	<0.001
	IIA	96.3 (85.8-99.1)		86.5 (73.7-93.3)	
	IIB	76.0 (58.5-86.9)		69.9 (52.8-81.8)	
	IIIA	75.4 (57.8-86.4)		60.8 (44.0-74.0)	
	IIIB	60.6 (29.4-81.4)		39.3 (14.5-63.6)	
	IIIC	61.9 (33.9-80.8)		43.9 (19.9-65.7)	

TNBC, triple negative breast cancer; BMI, body mass index; RFS, recurrence-free survival; OS, overall survival; CI, confidence interval.

**Table 3:** Results of Univariate and Multivariate Analyses

Covariate	OS				RFS			
	Univariate		Multivariate		Univariate		Multivariate	
	HR (CI 95%)	P	HR (CI 95%)	P	HR (CI 95%)	P	HR (CI 95%)	P
Age	1.03 (1.00-1.06)	0.09	1.01 (0.97-1.05)	0.58	1.01 (0.99-1.04)	0.28		
Subtype								
Luminal A	1	0.83			1	0.92		
Luminal B	0.72 (0.17-3.07)	0.66			1.00 (0.39-2.53)	0.99		
TNBC	1.14 (0.49-2.66)	0.76			1.15 (0.60-2.19)	0.68		
HER2 type	1.43 (0.54-3.78)	0.47			0.79 (0.31-2.00)	0.61		
TNM Stage								
I	1	0.001	1	0.003	1	<0.001	1	<0.001
IIA	1.21 (0.11-13.35)	0.88	1.53 (0.14-17.05)	0.73	5.79 (0.73-45.69)	0.1	5.88 (0.73-47.25)	0.1
IIB	8.92 (1.15-69.25)	0.04	8.12 (1.05-63.12)	0.045	10.91 (1.43-83.44)	0.02	10.96 (1.43-84.06)	0.02
IIIA	8.45 (1.09-65.65)	0.04	7.29 (0.94-56.89)	0.06	15.95 (2.13-119.70)	0.007	16.75 (2.22-126.13)	0.006
IIIB	21.24 (2.48-182.21)	0.005	30.18 (3.26-279.79)	0.003	39.24 (4.89-314.64)	0.001	36.94 (4.52-301.78)	0.001
IIIC	20.65 (2.53-168.53)	0.005	13.88 (1.70-113.74)	0.01	30.68 (3.88-242.78)	0.001	30.37 (3.82-241.43)	0.001
BMI (kg/m^2^)	1.10 (1.05-1.16)	<0.001	1.08(1.03-1.14)	0.002	1.05 (1.00-1.09)	0.04	1.05 (1.01-1.10)	0.02
Antidiabetic agent								
Metformin (REF: non-metformin)	0.12 (0.06-0.25)	<0.001	0.15 (0.07-0.32)	<0.001	0.24 (0.14-0.39)	<0.001	0.23 (0.14-0.40)	<0.001

TNBC, triple negative breast cancer; BMI, body mass index; RFS, recurrence-free survival; OS, overall survival; CI, confidence interval; REF, reference.

## Discussion

This study was designed to assess the role of metformin on the treatment of female breast cancer patients with type II DM. Our results showed a significant prognostic value of metformin compared to other antidiabetic agents in breast cancer patients which is in line with some previous references; the study of He et al at MD Anderson Cancer Center on 1,983 patients with the HER2 positive subtype of breast cancer showed that DM type II was an independent prognostic factor of poor OS in stage II or more of the disease, after adjustment for age, BMI, hormonal receptor status, nuclear grade, and stage. Another important finding of the mentioned study was that among breast cancer patients with DM type II, usage of metformin and thiazolidinediones were associated with improved OS (He et al., 2011). However, the study of Bayraktar et al from the same center did not show any significant advantage for diabetic metformin users in the adjuvant setting (Bayraktar et al., 2012); that subset of patients had poor prognostic factors of being obese, having TNBC pathologic subtype, or being black. 

A population-based study on older breast cancer patients (aged ≥66 years) was the first research to evaluate the time-dependent effects of metformin; even though a 5-year cumulative metformin usage led to 38% decrease in cancer-specific mortality, the HR was not significant (Lega et al., 2013). The authors discussed the limited proportion of patients with long follow-up time as the cause for lack of power and non-significant results. Another single-center study on 141 patients with type II DM revealed slightly higher OS and RFS rates in metformin users, yet the difference failed to attain the statistical level of significance (Oppong et al., 2014). Considering these controversies and lack of an agreement, some systematic reviews were performed in this field. The study of Xu et al, which analyzed 11 original studies, depicted a substantial role of metformin on overall survival of diabetic patients with breast cancer with HR of 0.35 (95% CI: 0.15-0.84) (Xu et al., 2015). Recent data from the Women’s Health Initiative also confirmed the benefit of metformin in terms of cancer-related mortality in breast cancer patients (Gong et al., 2016). Another meta-analysis, adding together 4 studies with and 4 other studies without significant results, concluded that the metformin effect on all-cause mortality of breast cancer was statistically significant (pooled HR: 0.63, P<0.001) (Cao et al., 2017). Similarly a bigger review confirmed the positive effect of metformin on OS of breast cancer patients (Yu et al., 2019).

Metformin has also been shown to be effective in malignancies of the gastrointestinal tract, lung, and prostate cancers (He et al., 2019; Chuang et al., 2018; Ramjeesingh et al., 2016; Xu et al., 2018; Zhang et al., 2018). Efficacy of metformin on different solid tumors, as well as its effects on both diabetic and non-diabetic patients, suggests an intertwined nexus of action in different organ systems. There are two main possible pharmacological pathways for metformin anti-cancer effects. The first is the insulin-dependent pathway which relies on insulin lowering function of the drug, thus decreasing proliferative capacity of the tumor (Heckman-Stoddard et al., 2017). The second is the insulin-independent pathway which acts directly on cancer cells (Gonzalez-Angulo and Meric-Bernstam, 2010). A key molecule affected by metformin in both pathways is 5’ adenosine monophosphate-activated protein kinase (AMPK) (Goodwin et al., 2009), which is a eukaryotic energy sensor (Alimova et al., 2009). Activation of AMPK causes inhibition of the mammalian target of rapamycin (mTOR) complex I, which in turn leads to inhibition of cell proliferation and biosynthesis of necessary components needed for cell growth and division (Martin-Castillo et al., 2010). An in vitro study on TNBC cell lines indicated that metformin has substantial activity on the induction of apoptosis and cell cycle arrest. However, in some experiments, metformin doses were above the therapeutic range. Given the fact that the metformin effects are time- and dose-dependent (Deng et al., 2012), the possibility for clinical application of these findings is limited (Liu et al., 2009). 

Another presumed mechanism for metformin action is alteration of the cyclin D and inhibition of cell cycle progression. Along this line, metformin has been shown to be effective in trastuzumab-resistant breast cancer cells (Liu et al., 2011; Vazquez-Martin et al., 2011). Nonetheless, the exact underlying mechanisms by which metformin induces anti-cancer capabilities are not completely known. The role of metformin in early-stage breast cancer was assessed in non-diabetic women in support of the significant effect of metformin on the reduction of tumor proliferation and increase of apoptosis (Niraula et al., 2012). On the other hand, some clinical window of opportunity studies that explored metformin’s effects on non-diabetic patients showed a significant reduction of Ki67 marker in accordance with its effect on glucose and insulin levels (Homeostasis Model Assessment score) (Sadighi et al., 2016). A basic research, through an in vitro study, using breast cancer cell lines of four different subtypes (Luminal-A, luminal-B, HER2 type and TNBC) in normoglycemic settings, failed to show added neoadjuvant value for metformin when compared to a taxane agent alone (Sadighi et al., 2014). Results of these two mentioned studies might be considered as another supporting evidence for indirect anti-cancer effects of metformin through interfering with insulin and insulin-like growth factors. 

The retrospective nature of the current study and comparatively small sample size are the main limitations to state a more solid conclusion about metformin’s potential on improving prognosis of cancer patients. In general, due to the diverse biologic nature of breast cancer and the manifold molecular mechanisms of metformin effect on different metabolic pathways, it seems that more specifically designed studies (e.g. randomized control trials) are needed to reach a more thorough understanding of its role on either diabetic or non-diabetic breast cancer patients. Well-tolerability, relatively low toxicity profile, availability and low-cost are the factors that should be considered for using metformin as adjuvant therapy. Late phase clinical trials are ongoing such as a Phase III Randomized Trial of Metformin vs. Placebo in Early Stage Breast Cancer sponsored by the Canadian Cancer Trials Group. As the clinical trials are the benchmark in medical research, we need to wait to reach a better understanding of metformin’s anti-cancer profile.
